# Effect of binocular vision during target shooting in archery

**DOI:** 10.1371/journal.pone.0294985

**Published:** 2023-11-30

**Authors:** Masakazu Hirota, Tatsuhiro Hanai, Takeshi Morimoto

**Affiliations:** 1 Department of Orthoptics, Faculty of Medical Technology, Teikyo University, Itabashi, Tokyo, Japan; 2 Department of Health and Sport Sciences, Graduate School of Medicine, Osaka University, Suita, Osaka, Japan; 3 Department of Advanced Visual Science, Graduate School of Medicine, Osaka University, Suita, Osaka, Japan; The Ohio State University, UNITED STATES

## Abstract

**Purpose:**

This study aimed to evaluate the difference between binocular and monocular vision and eye movements during the competition using video-oculography (VOG).

**Methods:**

Experiment 1 included 14 participants to evaluate differences in arrow convergence. Then, seven participants in Experiment 1 were randomly selected and included in Experiment 2, which evaluated eye movements during archery using VOG. The target used an 80-cm waterproof target face and was set at a distance of 30 m. All players shot the target 36 times using their bows and arrows. Experiments 1 and 2 evaluated the distribution of arrows in each score and the number of focus points, respectively, between binocular and monocular conditions.

**Results:**

The arrows, which include the area of 9 points, were significantly greater in the binocular condition (11.85 ± 5.04 shots) than in the monocular condition (9.36 ± 5.41 shots) in Experiment 1 (*P* = 0.047). The players focused on the target under both binocular and monocular conditions, although the players were switching off fixation between the target and shooting sight under the binocular condition in Experiment 2.

**Conclusion:**

These behaviors indicated that the players were trying to accurately shoot the target by exploring the distance between themselves and the target as a cue for depth perception.

## Introduction

Archery was first practiced at the Paris Olympics in 1900 and has been an official Olympic sport since 1972. Archery is simple: the archers draw the bow, aim at the target from 18 m (indoor) to 90 m (single round), and release the string. The score is determined by how well the arrows converge in the center of the target.

Experience and physical and mental training through repeated practice are essential to improve archery, as with any other sport [1]. Further, almost all sports require visual functions, including visual acuity, contrast sensitivity, and depth perception [2–4]. In particular, previous studies reported better sports performance under binocular vision, which provides stereopsis and depth perception, than under monocular vision, which is different in basketball and table tennis players [3, 4]. Conversely, stereopsis in soccer players does not differ between professionals and amateurs. These results indicate various advantages of binocular and monocular vision depending on the sports category. Depth perception and stereopsis are expected to be highly essential for shooting distant targets in archery.

**Fig 1:**
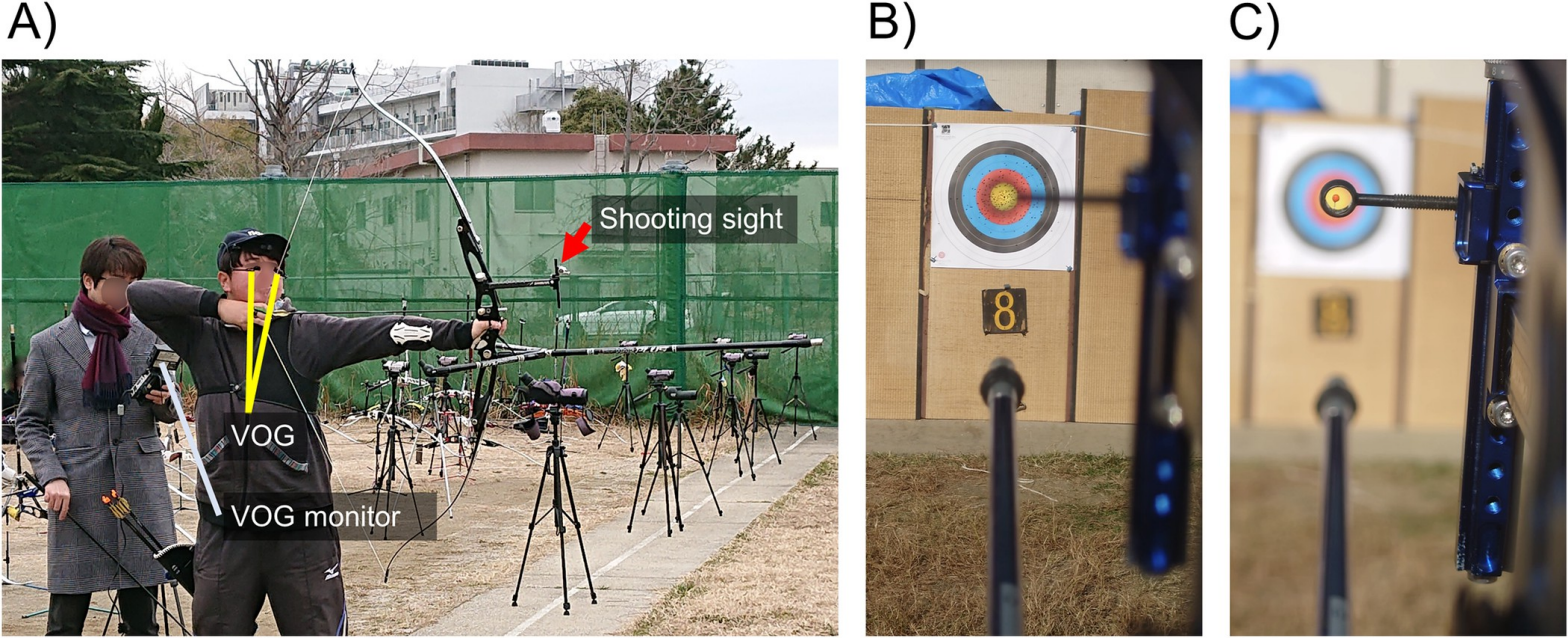
Experimental scene of archery in this study (A) and hypothetical focus position of participants (B and C). This study used VOG to record eye movements during archery competitions (A). We hypothesized that participants perceived distance by alternately viewing the distant target (B) and the nearby shooting sight (C). VOG: video-oculography.

Earlier studies reported that depth perception is valid at a distance of 40 m [5, 6]. Therefore, binocular vision may be more advantageous than monocular vision in archery, as the target is located 30 m from the archer. However, the advantage of binocular conditions in archery is unknown because of the complex eye movements during the competition. Most players have shooting sights attached to their bows’ tips and are looking at both the distant target and near shooting sight during the competition (Fig 1A). Hence, there are two states: viewing the target through the shooting sight (Fig 1B) and looking at the center of the shooting sight and aligning it with the target (Fig 1C). We hypothesized that participants would acquire a sense of perspective and increase their archery scores by binocularly viewing the distant target and the nearby shooting sight.

Therefore, the player’s gaze should be recorded to assess the advantage of the binocular conditions. This study used video-oculography (VOG) to measure the gaze position during archery competitions (Fig 1A) and assess the difference in gaze position and score under binocular and monocular conditions.

## Methods

### General procedures

An ophthalmologist (T.M.) assessed all study participants. They had no ocular disease, such as strabismus or amblyopia, except for myopia. Further, participants with myopia were wearing glasses or contact lenses. The ocular dominance was measured using a hole-in-card test.

This investigation was conducted following the principles of the World Medical Association Declaration of Helsinki. All participants provided written informed consent after explaining the nature and possible risks of the study to them. The Institutional Review Board approved the experimental protocol and consent procedures of Osaka University (approval no.17451-2).

### Target of archery

The target used an 80-cm waterproof target face (JVD Archery, Nieuwkuijk, The Netherlands) and was set at a distance of 30 m from a player. All players shot the target 36 times using their bows and arrows. Three shots are attempted in fewer than 2 min in archery competitions, and 36 shots are attempted in each round. Therefore, this study changed and crossover the conditions for the first and second rounds to reduce the fatigue effect of shooting arrows.

### Covering one eye

**Fig 2:**
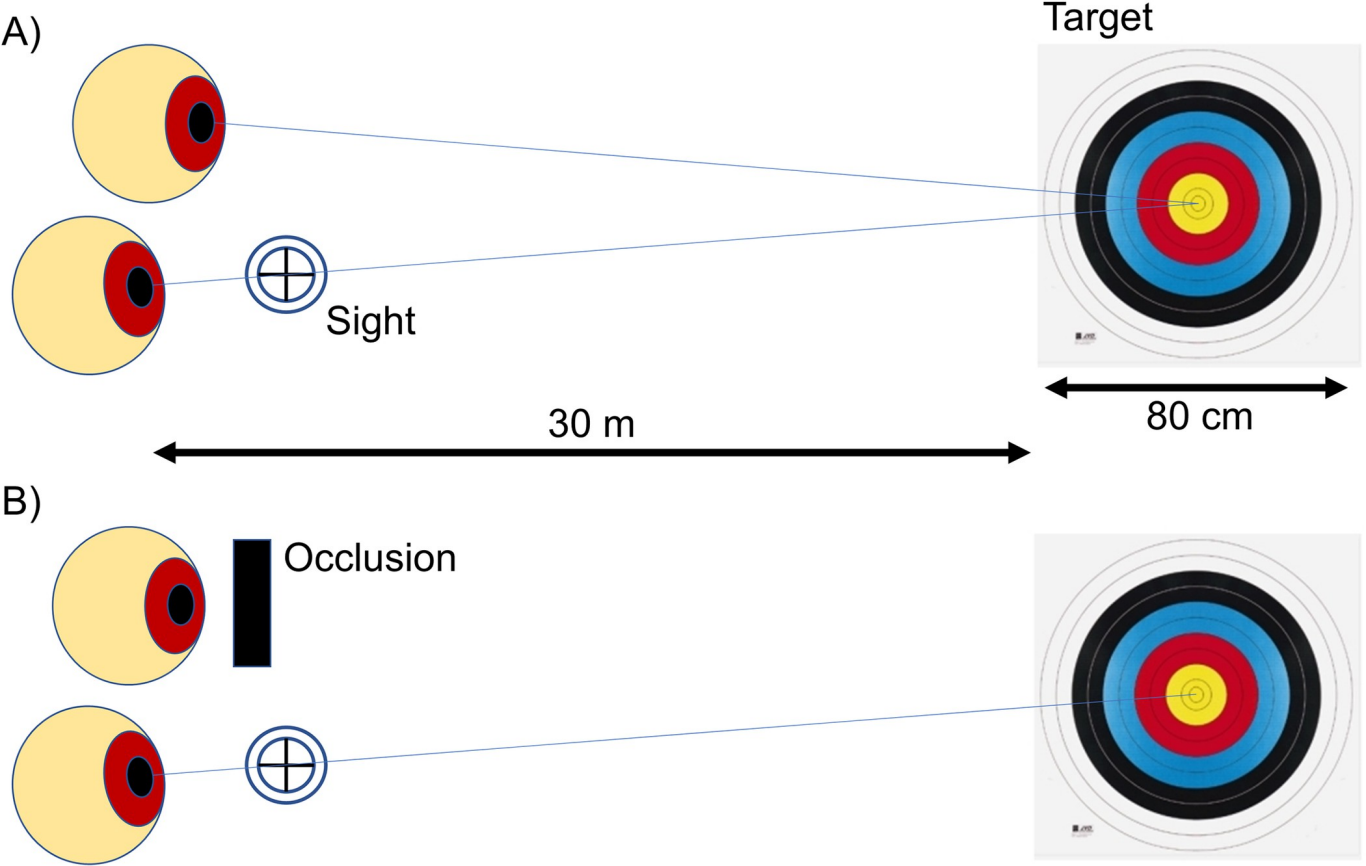
Binocular (A) and monocular (B) conditions in the experiment. The players shoot 36 times under binocular or monocular conditions in Experiments 1, 2, and 3.

The participants covered one eye to assess the difference between binocular and monocular conditions. We checked beforehand which eye the participants used to view the shooting sight. All participants used their right eye when looking at the shooting sight, which was also the motor-dominant eye. The nondominant eye was completely covered with an eye patch (Eye-patch A2, Kawamoto Corp., Osaka, Japan) to avoid binocular rivalry (Fig 2).

### Score counting

**Fig 3:**
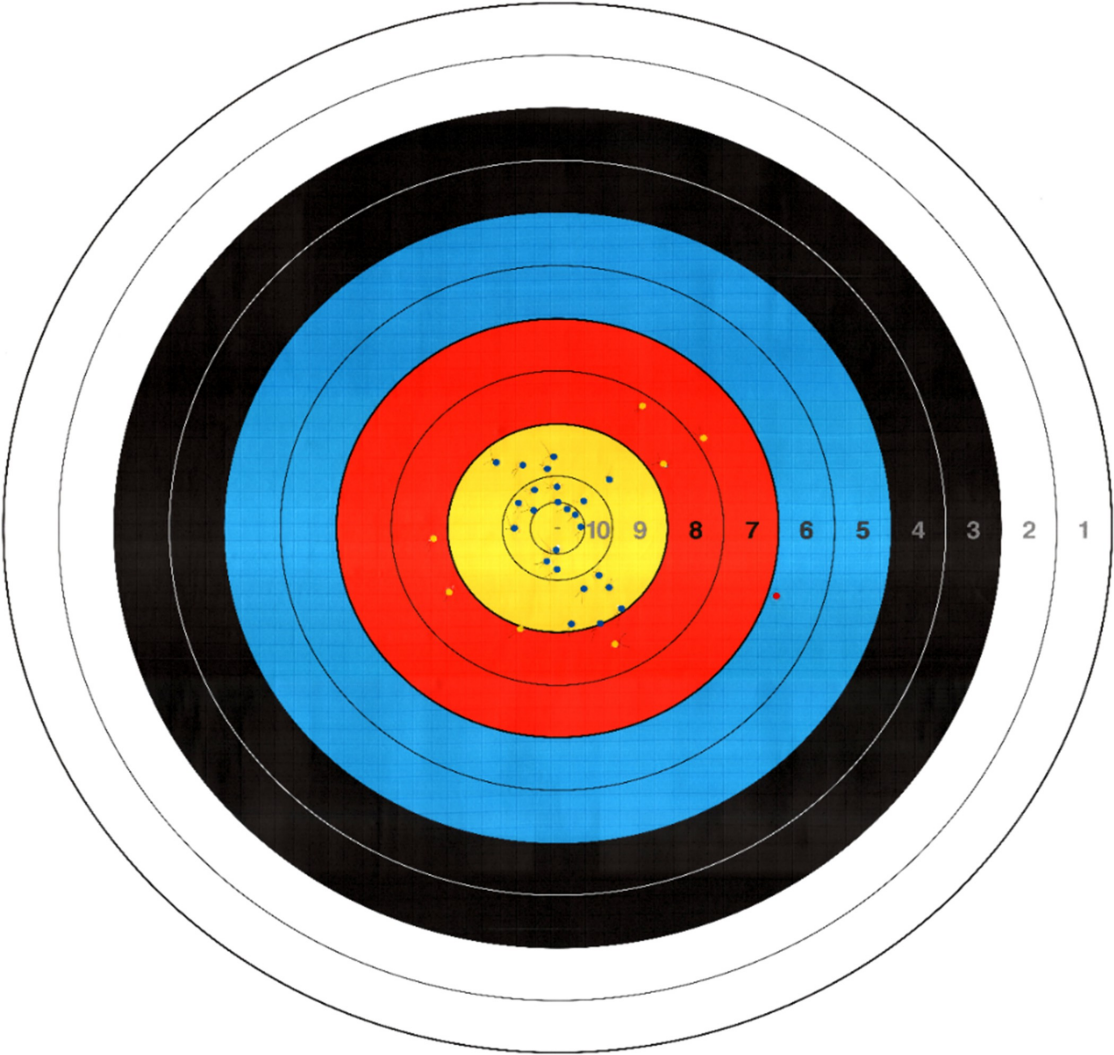
A target image with the labeled hole points. The hole points were created by the arrows and labeled by solid seals (blue, yellow, and red dots). The target was scanned and converted to a resolution of 1,718 × 1,628 pixels.

The examiner labeled the hole points on the target with the arrows using solid seals (MyTack, Nichiban Co., Ltd.; Tokyo, Japan). The target was scanned and converted to a resolution of 1,718 × 1,628 pixels and jpeg compression (Fig 3). Subsequently, ImageJ calculated the horizontal and vertical positions of solid seals by ImageJ (Wayne Rasband, NIH).

The center of the target was defined as horizontal at 0.0° and vertical at 0.0°. The target’s right- and upper halves were defined as the positive sides, and the left- and lower halves as the negative sides. The direction of horizontal convergence of the arrows, in cases where the left eye is the dominant eye, is the opposite of that seen in cases where the right eye is dominant. Therefore, the horizontal axis under the monocular condition when the left eye is dominant was reversed for data normalization.

## Experiment 1

### Methods

Experiment 1 evaluated the accuracy between binocular and monocular conditions. Experiment 1 included 14 players belonging to the archery club at Osaka University (mean age [± standard deviation] 20.7 ± 1.5 years). Participants were randomly categorized into two groups with matched ages, average scores in the last three competitions, and athletic history. One group first shot 36 times under the binocular condition and subsequently shot 36 times under the monocular condition, and the eye not focused on the shooting sight was covered. Another group first shot 36 times under monocular conditions, followed by 36 shots under binocular conditions.

### Data analysis

The center of gravity was calculated from the average of the 36 horizontal and vertical positions where the arrows stuck, for each player under binocular and monocular conditions, respectively.

The kernel density map for the horizontal and vertical distribution of arrows was analyzed using Python 3.8.5 on Windows 10 (Microsoft Co., Ltd., Redmond, WA, USA) with the following libraries: Matplotlib 3.3.2, Numpy 1.18.5, Pandas 1.1.3, and Seaborn 0.11.0.

The number of arrows in each score was recorded to evaluate their distribution.

### Statistical analysis

The Wilcoxon signed-rank test with the Shapiro–Wilk test analyzed the differences in the total score and the center of gravity. The paired t-test with the Shapiro–Wilk test analyzed the distribution of arrows in each score between binocular and monocular conditions.

The Statistical Package for the Social Science (SPSS) version 26 (IBM Corp., Armonk, NY, USA) determine the significance of the differences, and a *P*-value of <0.05 was considered significant.

### Results 1

Table 1 presents the characteristics of the two groups. The mean age (*P* = 0.48), mean score in the last three competitions (*P* = 0.96), and athletic history (*P* = 0.23) were not significantly different between the two groups.

**Table 1:** Characteristics of players.

	B to M	M to B	*P*-value
Number of players	7	7	−
Gender ratio (male: female)	7: 0	4: 3	0.025 †
Age (year)	20.3 ± 1.0	21.1 ± 1.8	0.48
Average score (points)	334.0 ± 17.6	336.0 ± 12.7	0.96
Athletic history (years)	5.0 ± 3.8	3.0 ± 1.6	0.23

B to M, the group that shot 36 times under binocular conditions and 36 times under monocular conditions; M to B, the group that shot 36 times under monocular conditions and 36 times under the binocular conditions.

†: Fisher’s exact test. Other *p*-values were analyzed by paired t-test.

**Fig 4:**
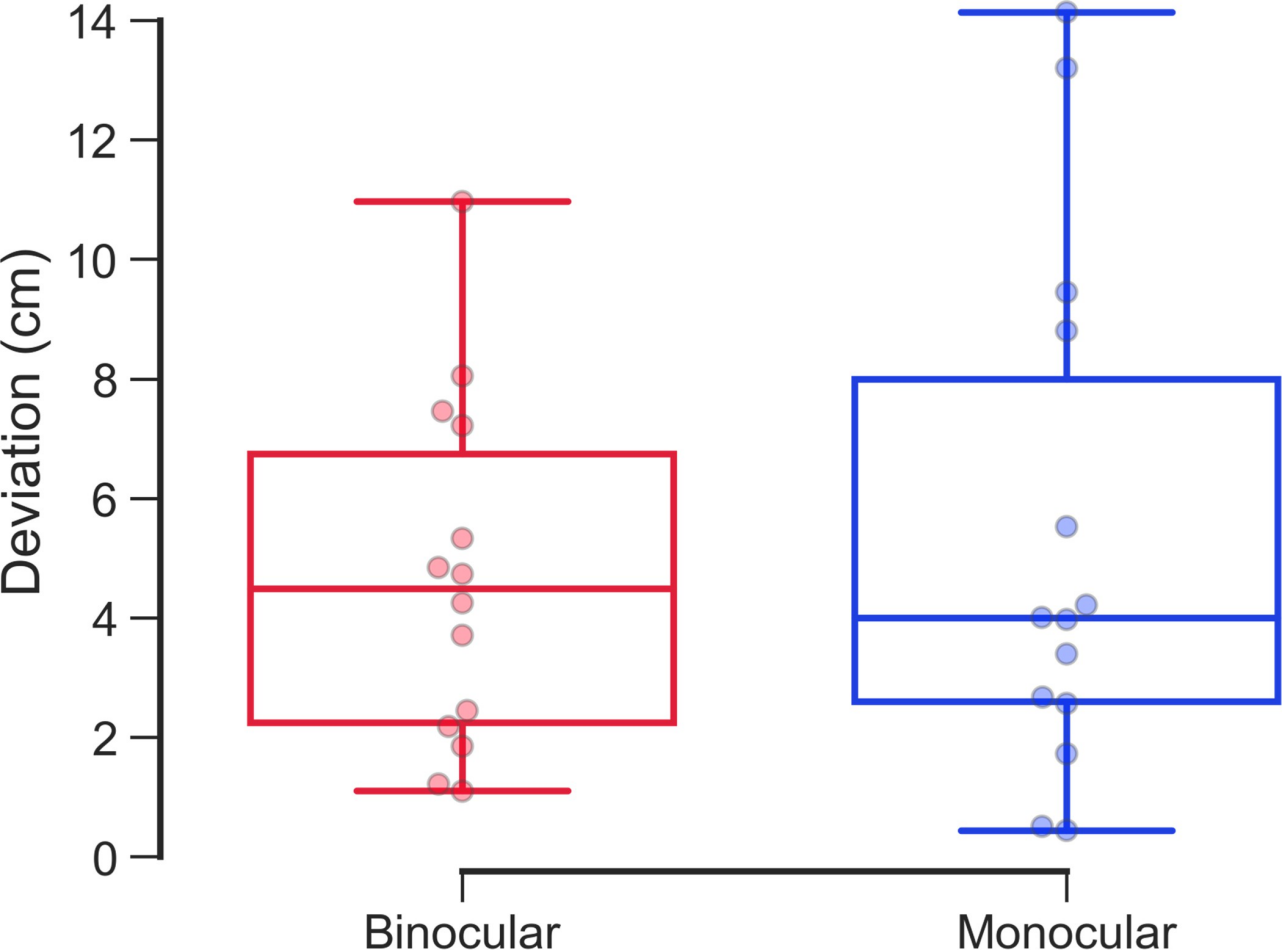
Comparison of the center of gravity in 36 shots between binocular and monocular conditions. The red and blue boxplots with dots indicate the average center of gravity in 36 shots under the binocular and monocular conditions, respectively. The center of gravity in 36 shots was not significantly different between the binocular and monocular conditions.

The total score was not significantly different (*P* = 0.22, Wilcoxon signed-rank test) (Table 2) as well as the center of gravity in 36 shots between the binocular and monocular conditions (*P* = 0.83, Wilcoxon signed-rank test) (Fig 4) (Table 3).

**Table 2:** Total score between binocular and monocular conditions.

		Shapiro–Wilk test	
Condition	Total score (points)	*W*-value	*P*-value
Binocular	257.6 ± 70.7	0.85	0.017
Monocular	250.9 ± 72.5	0.1	<0.001

**Table 3:** Center of gravity in 36 shots between binocular and monocular conditions.

		Shapiro–Wilk test	
Condition	Center of gravity (cm)	*W*-value	*P*-value
Binocular	4.67 ± 2.81	0.94	0.39
Monocular	5.33 ± 4.24	0.88	0.048

**Fig 5:**
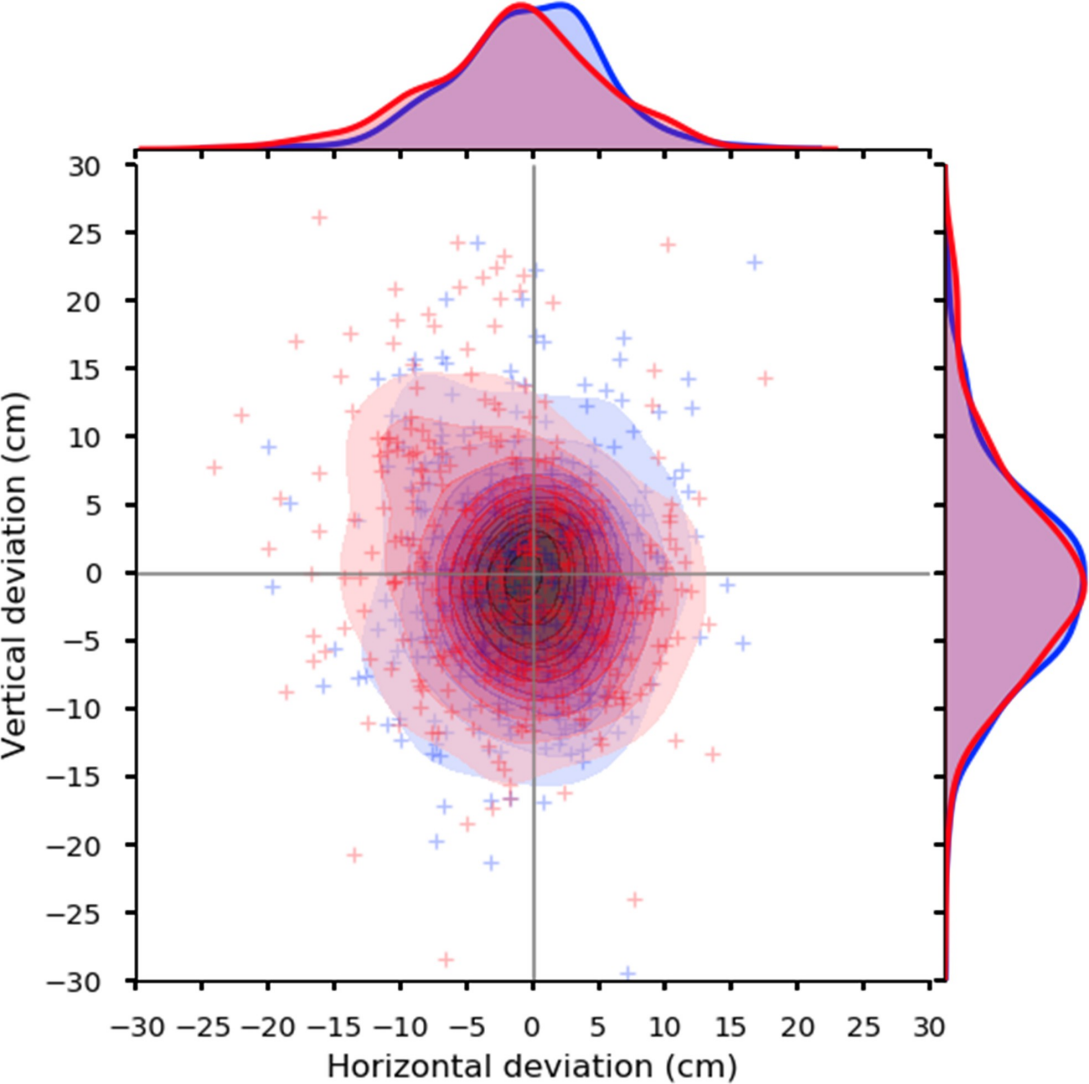
Distribution of arrows in all players under binocular (red) and monocular (blue) conditions. The red and blue crosses show where the arrow hit the target under binocular and monocular conditions in all players, respectively. The red and blue contour lines indicate the kernel density map in the distribution of arrows in all players under binocular and monocular conditions, respectively. The red and blue curves on top and right indicate the arrows’ horizontal and vertical distribution in all players under binocular and monocular conditions, respectively. The convergence of the arrow in the horizontal direction was closer under the binocular condition compared with the monocular condition.

**Fig 6:**
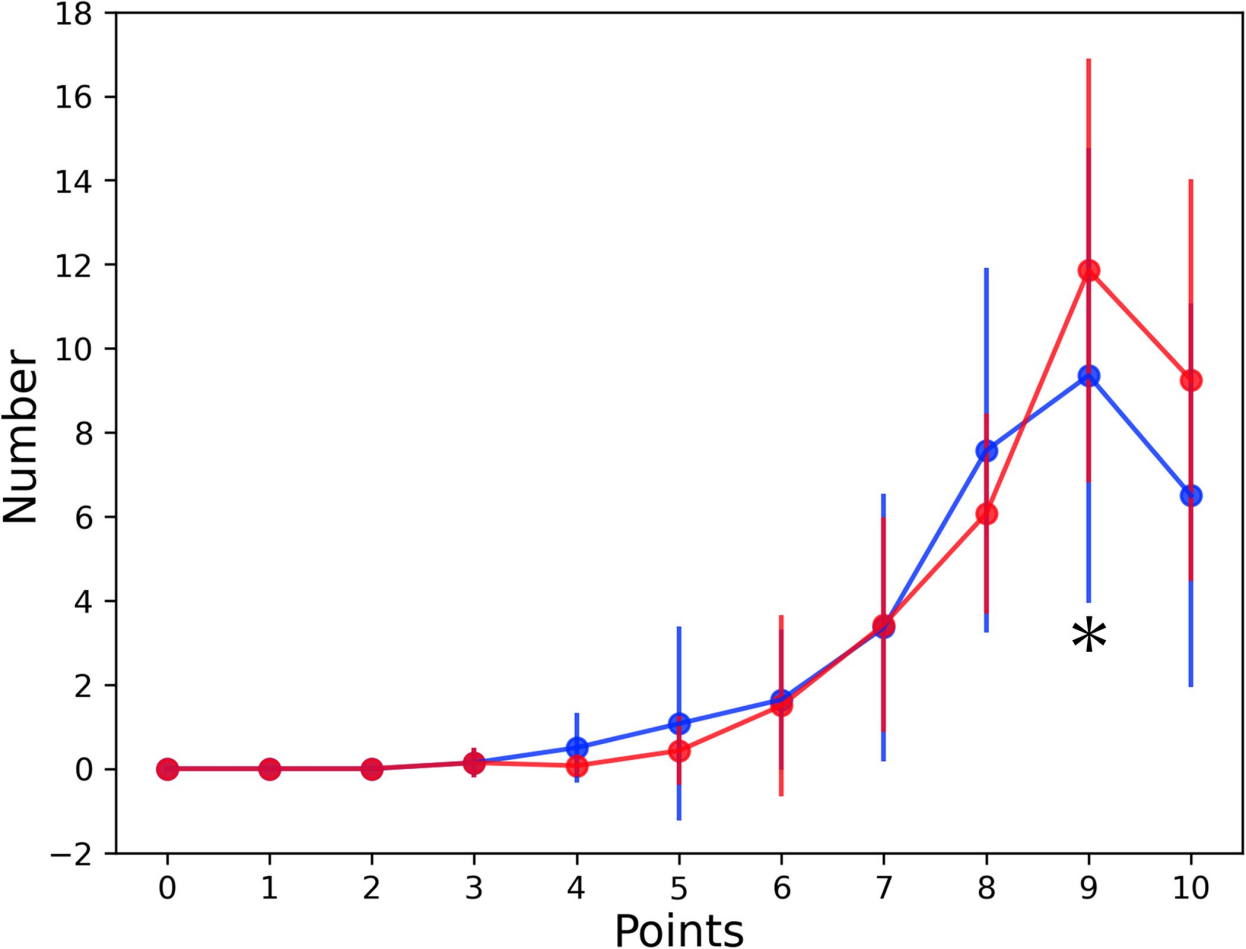
Differences in score distribution between binocular and monocular conditions. Red and blue dots indicate the average number of arrows in each score under binocular and monocular conditions, respectively. Red and blue lines indicate standard deviations in each score under binocular and monocular conditions, respectively. The numbers of arrows in points 4, 5, 6, and 8 were lower under the binocular condition than the monocular condition, and the number of arrows in point 9 was significantly greater under the binocular condition than the monocular condition. *: *P* = 0.047.

Fig 5 shows the distribution of arrows in all players under binocular and monocular conditions. The convergence of the arrow in the horizontal direction tended to be close under the binocular condition compared with the monocular condition. The comparison of binocular and monocular conditions at each score revealed a significantly greater number of arrows, which were located in the area of 9 points, under the binocular condition compared with the monocular condition (*P* = 0.047). Conversely, the numbers of arrows in points 4, 5, 6, and 8 were lower under the binocular condition than the monocular condition (Fig 6 and Table 4).

**Table 4:** Distribution of arrows in each score between binocular and monocular conditions.

Point	Binocular	Monocular	*P*-value
0	0 ± 0	0 ± 0	−
1	0 ± 0	0 ± 0	−
2	0 ± 0	0 ± 0	−
3	0.14 ± 0.35	0.14 ± 0.35	0.99
4	0.07 ± 0.26	0.50 ± 0.82	0.082
5	0.43 ± 0.82	1.07 ± 2.31	0.28
6	1.50 ± 2.16	1.64 ± 1.67	0.67
7	3.43 ± 2.56	3.36 ± 3.18	0.92
8	6.07 ± 2.37	7.57 ± 4.34	0.151
9	11.86 ± 5.04	9.36 ± 5.41	0.047
10	6.64 ± 4.78	6.50 ± 4.56	0.89

The peak of the arrow distribution was more shifted to the center under binocular vision than under monocular vision. This difference may have caused fewer arrows located in 4-, 5-, 6-, and 8-point areas and a significantly higher number of arrows located in the 9-point area under binocular vision than under monocular vision.

## Experiment 2

### Methods

Experiment 2 recorded the players’ eye movements during a competition using the VOG (EMR-9, NAC Image Technology Inc., Tokyo, Japan) (Fig 1A). The VOG device determined the eye positions by detecting the corneal reflex and pupil center by reflecting the near-infrared light with a sampling rate of 240 Hz. The measurement error was 0.2°–0.5° (interquartile range) at a distance of 1.0 m. The scene camera recorded real scenes (resolution: 640 × 480 pixels; angle of view: ±44° from the center of the scene camera) with a sampling rate of 29.97 Hz. The images obtained by the eye and scene cameras were sent to the controller. The controller computes the gaze position of both eyes from the corneal reflex and pupil center. Subsequently, the gaze positions are merged with the real scenes at a delay of ≤52 ms. VOG outputs each eye to record eye position and pupillary diameter in a comma-separated values file and the video recorded by the scene camera in an M4F file. VOG combines the spatial connection between the scene and eye cameras through gaze calibration.

All players underwent a calibration test to change their gaze position under binocular conditions with fully corrected glasses before undergoing the eye movement test. The calibration distance of 120 cm was determined by setting the interpupillary distance to 60 mm so that eye movements between the target and the shooting sight could be equally assessed. The center of the scene camera was adjusted to approximate the target’s center at 30 m with the player’s simulated state of the pulled arrow. All players were asked to fixate a cross target that moved to nine positions during the calibration: (horizontal of 0.0°, vertical of 0.0°), (0.0°, 15.0°), (15.0°, 15.0°), (15.0°, 0.0°), (15.0°, −15.0°), (0.0°, −15.0°), (−15.0°, −15.0°), (−15.0°, 0.0°), and (−15.0°, 15.0°), respectively. The center of the calibration plate was defined as 0°, the right- and upper halves of the screen as the positive sides, and the left- and lower halves as the negative sides.

Seven players who participated in Experiment 1 with a mean age of 19.7 ± 0.7 years were randomly selected to participate in Experiment 2. The refractive error under refractive correction that the players usually compete in was measured using Spot Vision Screener^TM^ (Welch Allyn, Skaneateles Falls, NY) in an outdoor archery arena. Participants shoot 36 times under binocular and monocular conditions randomly. The eye, which was not fixated on the scope, was covered.

### Data analysis

Data were excluded when the change in pupil diameter was >2 mm/frame due to blinking [7] and the missing values were replaced with a linearly interpolated value calculated from an algorithm written in Python 3.6.5. The horizontal and vertical correct eye positions were analyzed.

The right eye data were obtained, and Python 3.8.5 on Windows 10 (Microsoft Co., Ltd. Redmond, WA, USA) calculated the kernel density map for horizontal and vertical eye positions during the shooting. A value of 0° indicated the center of the scene camera. Positive values represented right- or upper halves, whereas negative values represented left or lower halves.

The horizontal focus point in each player was estimated from the center of the density map. The primary focal point was the most negative value if the horizontal focus point detects more than two points.

### Statistical analysis

Fisher’s exact test analyzed the differences in the number of focus points between binocular and monocular conditions. The simple linear regression analysis examined the correlation of the primary focus point between binocular and monocular conditions. The Wilcoxon signed-rank test analyzed the pupil diameter for 1 s just before shooting an arrow between binocular and monocular conditions.

IBM SPSS version 26 was used to determine the significance of the differences, and a *P*-value of <0.05 was considered significant.

### Results 2

Each participant’s mean refractive error was −0.66 ± 0.68 D in the right eye and −0.54 ± 1.16 D in the left eye (Table 5).

**Table 5:** Ocular refraction during an archery competition.

	RE		LE	
ID	SE	SCL	SE	SCL
1	−0.625	−7.00	−1.00	−7.00
2	−0.75		−1.00	
3	−0.375	−1.75	−0.875	−1.25
4	−2.25		−2.50	
5	−0.125	−4.00	−0.375	−4.50
6	−0.375	−1.75	−0.25	−1.75
7	−0.125		1.50	

RE: right eye; LE: left eye; SE: spherical equivalent; SCL: soft contact lens.

**Fig 7:**
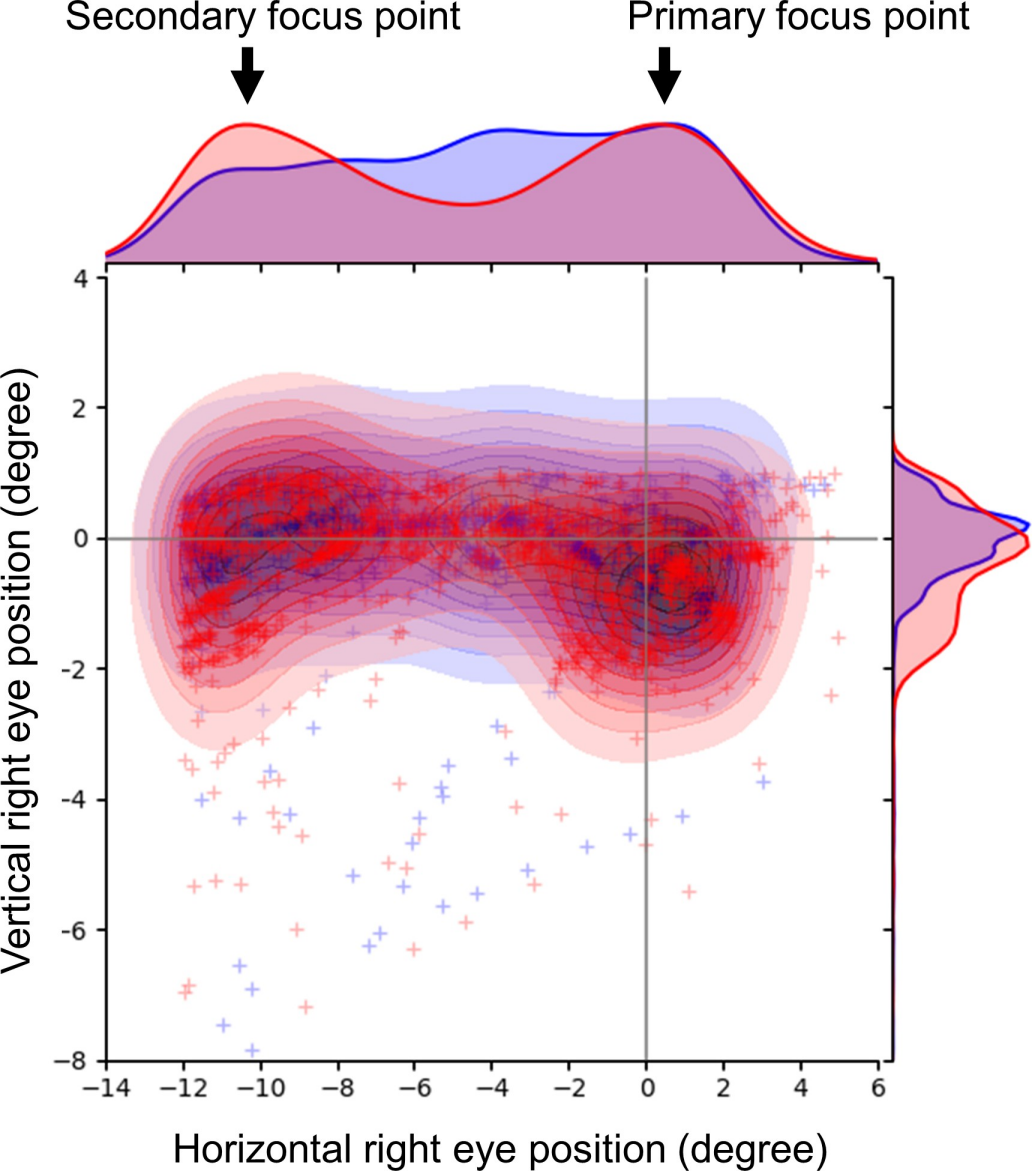
Representative eye movements in shooting the arrows. The red and blue crosses indicate the eye positions in 36 shots under binocular and monocular conditions, respectively. The red and blue contour lines indicate a kernel density map in the distribution of eye positions in 36 shots under binocular and monocular conditions, respectively. The red and blue curves on the top and right show the horizontal and vertical distribution of eye positions in 36 shots under binocular and monocular conditions, respectively. Participants often shoot the arrows by looking at the target and sight using binocular vision.

Fig 7 shows the distribution of arrows for the representative player (player 7) under binocular and monocular conditions. Five players under the binocular condition and two players under the monocular condition see both the target (primary focus point) and shooting sight (secondary focus point) (*P* = 0.051) (Table 6).

**Table 6:** Frequency of switching fixation in the binocular and monocular conditions.

	Switched the fixation	No switching fixation
Binocular	5	2
Monocular	2	5

**Fig 8:**
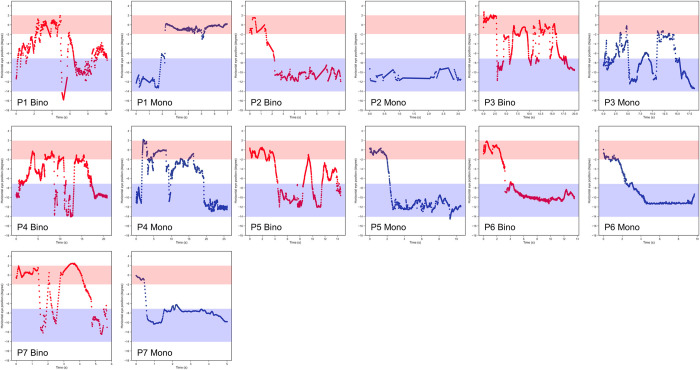
Eye movements in the one-shot under binocular (red) and monocular (blue) conditions. The red and blue zones indicate when looking at the target and shooting sight, respectively. Five players switch between the target and sight in the binocular condition.

Fig 8 depicts the one-shot’s representative eye movements under binocular and monocular conditions. The player switches fixation between the target and shooting sight in the binocular condition until releasing the arrow. In contrast, the player in the monocular condition first looks at the target and then at the shooting sight without switching fixation until the arrow is released.

**Fig 9:**
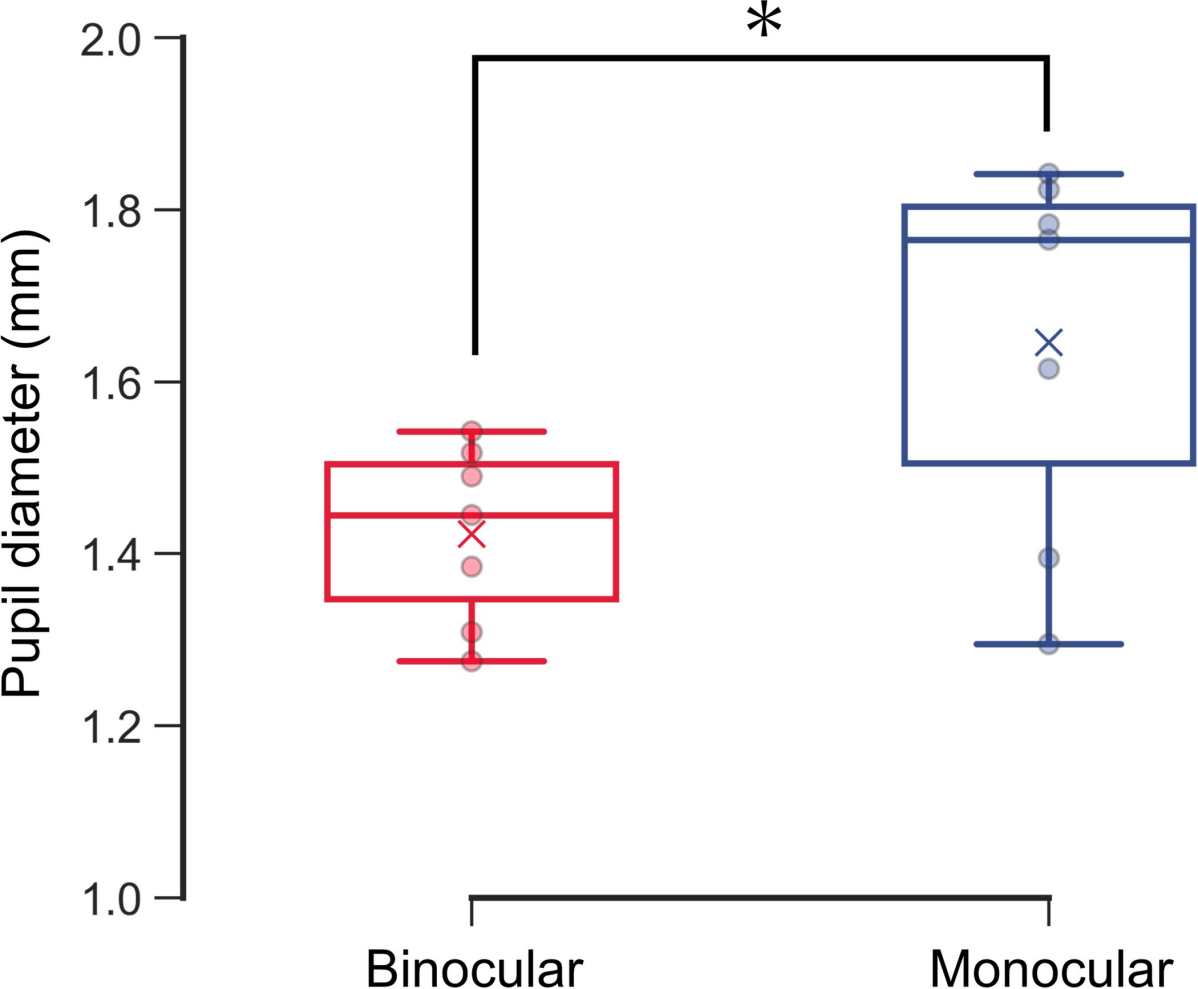
The pupil diameter for 1 s just before shooting an arrow. The red and blue boxplots with dots indicate the pupil diameter under binocular and monocular conditions, respectively. The red and blue crosses indicate the mean values of all players. Pupil diameter was significantly smaller under binocular conditions than under monocular conditions. *: *P* = 0.018, Wilcoxon signed-rank test.

The pupil diameter was significantly smaller under binocular conditions (1.42 ± 0.01 mm) than under monocular conditions (1.64 ± 0.02 mm) (*P* = 0.018) (Fig 9).

## Discussion

This study evaluated the advantage of binocular vision in the archery competition. Experiment 1 revealed significantly different in the center of gravity in all 36 shots between the binocular and monocular conditions (Fig 4). However, the convergence of the arrows in the horizontal direction was closer under the binocular condition than with the monocular condition (Fig 5). The number of arrows which are located in the area of 9 points, were significantly greater under the binocular condition than under the monocular condition. Furthermore, the number of arrows located in the area of 9 points was reduced under the binocular condition (Fig 6 and Table 4). These findings indicate that aiming at the center of the target is easier with binocular vision than with monocular condition.

However, our results are inconsistent with previous studies by Strydom [2]. They reported better convergence of the arrow in the monocular condition than in the binocular condition in traditional archery, which does not use the shooting sight. The definition of monocular vision differs between the present study and earlier studies. The present study created a monocular vision condition by completely covering one eye, whereas earlier research defined monocular vision as a person viewing a target with one eye while both eyes were open.

Most participants in Experiment 2 switched the fixation between the target and the shooting sight under the binocular condition (Figs 7 and 8). However, a few players switched the fixation between the target and the shooting sight under the monocular condition, and they looked at the shooting sight most of the time until they shot arrows (Fig 8). These results indicated that the binocular condition involves looking at both the target and shooting sight to adjust the position of the arrow to accurately hit the target. Piano et al. reported that degrading binocular fusion and stereoacuity, which are related to depth perception, significantly affect performance in certain fine visuomotor tasks [8]. Depth perception is better under binocular conditions than under monocular conditions based on binocular disparity [9, 10]. Physiologic diplopia occurs with the fixation on distant and nearby objects [11, 12]. The participants switched fixation between the target and shooting sight, and they obtained depth perception via binocular disparity. Further, the effects of the visual field and binocular rivalry caused better arrow convergence under the binocular condition than under the monocular condition. The horizontal visual fields in the binocular and monocular conditions are reported as >180 degrees and approximately 100 degrees, respectively [13]. We consider a minute effect on the field of view because the size of the target was 1.53 degrees. Additionally, we consider a negligible effect of binocular rivalry in this study because one eye was completely covered and visual information was blocked.

The pupil diameter in all subjects was significantly smaller under binocular conditions than under monocular conditions (Fig 9). This finding indicates that participants fixated on the shooting target before releasing the arrow under binocular conditions because convergence, accommodation, and miosis are interlinked as a near reflex. We determined that the participants were aiming by alternately looking at the target and the center of the sight based on their eye movements and pupillary response. The participants need to have a visual acuity of 0.218 logMAR or better to see the center circle of the target at 30 m. All study participants performed archery games with refractive correction [14]. Furthermore, the participants obtained binocular disparity under binocular conditions by switching the fixation between the distant target and near shooting sight (Fig 8). These findings were consistent with Gillam et al., who reported proper depth perception at a distance of over 40 m with both motion and binocular disparity [15].

Our findings revealed that binocular vision in archery provides some benefits to athletes. As a limitation, this study was conducted on amateur athletes in university students and not on professional archery athletes. Wei et al. reported that the cortical thickness of the superior temporal sulcus (STS) was significantly greater in professional athletes than in amateur athletes in diving [16]. The STS involves the perception of biological motion [17]. Faubert reported that professional athletes could learn to process complex dynamic visual scenes compared to amateur athletes [18]. Thus, professional athletes may show different results between the binocular and monocular conditions in archery.

Another limitation is that the eye position assessment included relative and not absolute values. The eye positions using VOG affect head posture because the human eye shifts to counter the position related to the head posture by the oculocephalic reflex. The participants underwent a calibration test under their simulated state of the arrow being pulled to prevent the affection of the head posture. However, the participants’ head positions changed during the competition. The participants’ head positions slightly altered with each arrow shot, causing their faces to turn and their heads to tilt. Consequently, a little alteration was made in the eye position’s origin.

## Conclusions

This study investigated the difference between binocular and monocular vision in archers. The overall score is commonly used as an evaluation parameter in sports vision studies. This study developed a method for determining the position of individual arrows and measuring eye movements during archery competitions using VOG. In particular, the method for determining the position of individual arrows could detect minute differences that could not be captured by the overall score. We believe that our method can be used to optimize the conditions although the approach to improving archery scores differs among individuals.
